# The Extraction of Living Pulps from Teeth under the Influence of Cocaine Anæsthesia Produced with the Aid of Mechanical Pressure

**Published:** 1900-09

**Authors:** Wilson Zerfing

**Affiliations:** Philadelphia


### International Dental Journal.

Vol. XXI. September, 1900. No. 9.

### Original Communications.[1]

### THE EXTRACTION OF LIVING PULPS FROM TEETH UNDER THE INFLUENCE OF COCAINE ANÆSTHESIA PRODUCED WITH THE AID OF MECHANICAL PRESSURE.[2]

BY WILSON ZERFING, D.D.S., PHILADELPHIA.

Mr. President, Members of the Academy, Ladies and Gentlemen,—The extraction of the tooth-pulp has for many years been recognized as a necessity in dentistry, and with it also the necessity of some means of performing the operation painlessly.

As early as 1836 Dr. J. R. Spooner, of Montreal, has been credited with introducing arsenous acid as an agent, or, I might almost say, *the* agent, to render a pulp insensible, and although he first used it alone, it has since been combined with other agents in various formulæ, and is used largely even at the present day.

Although perhaps more pulps have been removed from teeth after first having been treated with arsenic than with the combined use of all other means, I think the profession has always felt that arsenical devitalization of pulps for their extirpation has, as a rule, not been as satisfactory as might be desired.

[1] The editor and publishers are not responsible for the views of authors of papers published in this department, nor for any claim to novelty, or otherwise, that may be made by them. No papers will be received for this department that have appeared in any other journal published in the country.

[2] Read before the Academy of Stomatology, April 24, 1900.

With the advent of cataphoresis we had hoped to be able shortly to entirely abandon the use of this drug, but for various reasons cataphoresis has not proved the boon it was hailed to be, and consequently it was used for pulp-extraction in comparatively few cases only, even in the hands of those possessed of the cataphoric apparatus. In the first place, the requirement of the necessary appliances rendered its use not generally popular, owing, perhaps, largely to the time necessary to properly adjust the same and the uncertainty of its action, especially in the hands of those not careful as to details; although there can be no denying the fact of its utility in rendering the dental pulp insensible to pain under proper and careful manipulation.

In reading a copy of the May, 1899, issue of *Items of Interest*, my attention was attracted by an article appearing in "The Editor's Corner," calling attention to the fact that, "Some certain person, or, rather, some uncertain person, as his identity had not been known, was travelling through the West selling 'a method of painlessly removing pulps,' and charging twenty-five dollars for the secret." In the same article also was divulged the secret, as given by a correspondent, which no doubt many of you have learned.

The simplicity of the treatment seemed to offer such marked advantages over any other means with which we were acquainted that it was considered worthy of a trial, and the result obtained proved most astonishing.

I commenced by using the formula given by Dr. Mitchell, of formalin, one part; absolute alcohol, five parts; and pulverized cocaine crystals, taken up with a piece of spunk previously saturated in the above solution, being careful in each instance to use a piece of spunk no larger than the opening of the exposure. The procedure followed was that outlined in the referred-to article.

At a later period, placed in the position of being without formalin, it was determined to try the alcohol alone with the cocaine, and the result proved quite as successful as with the addition of formalin, so ever since it has proved very satisfactory without the latter.

Following is a summary report of cases thus treated, showing the diversified conditions and positions in which the treatment is applicable, for many of which we are indebted to the clinic at the University of Pennsylvania.

In a few of the cases you will notice a slight digression from the original in the substitution of eucaine for cocaine, and vapocaine for alcohol, the manner of applying, however, being the same, as well as the effect.

*October 11.*—Mr. B., aged about thirty-eight. Inferior left second molar, cavity involving the larger part of the occlusal surface; pulp exposed, but conditions apparently favorable for capping; cavity treated, pulp capped, but tooth filled only temporarily with gutta-percha. The treatment proved unsuccessful, and devitalization became necessary. Used eucaine by crushing part of a tablet on a glass slab, taking it up with the spunk previously dipped in vapocaine, and proceeding with the pressure as suggested, all sensation disappearing in a few moments; when on opening the pulp-chamber with a bur, and an attempt made to enter the canals, the chamber was found filled with pulp-nodules, the removal of which caused considerable trouble, as they were quite difficult to dislodge, and by the time the canals could be entered without obstruction an application of the anæsthetic was again made to the pulp at the entrance to each canal, when its removal was accomplished without pain. Four days later the canals were filled with oxychloride of zinc, the tooth doing good service.

*November 13.*—Mrs. B., aged about fifty-three, applied for the repair of a bridge previously anchored to the inferior left first bicuspid by a very thin, soft, open-faced shell crown, and extending back to the second molar. The fixture had done service a little less than nine months, when the bicuspid shell had gone to pieces. The tooth used for anchorage had been very imperfectly trimmed; in fact, no grinding had been done practically, which necessitated considerably more cutting before a satisfactory repair could be undertaken; the tooth, however, was so extremely sensitive to even ordinary conditions that extraction of the pulp was decided upon. An opening was drilled through the occlusal surface, and with the aid of vapocaine the pulp exposed, when an application of eucaine was made directly to it, as in the previous case, and the pulp extracted entirely without pain, when all trouble as to sensitiveness was at an end. The canal was subsequently filled with oxychloride of zinc.

*November 14.*—Mr. K., aged thirty. Inferior left first bicuspid, mesial cavity, pulp exposed; sensitive and ached at times. Applied cocaine and, as solvent, ordinary commercial alcohol; the pulp was extracted with very slight pain, and after properly dressing, the canal was immediately filled with oxychloride of zinc, and the cavity filled four days later with tin and gold.

*November 14.*—Miss L., aged nineteen. Inferior right first bicuspid, mesial cavity; also inferior left first and second bicuspids, cavities approximating; all three pulps exposed and in an inflamed condition, and apparently all three aching at the time of visit. Applied cocaine and alcohol, and extracted all three pulps at the same sitting, the first painlessly, the second almost so, while the third, which had a very small exposure, required a second application, which produced complete anæsthesia. The canals were dressed and filled at a subsequent sitting.

*November 15.*—Mr. R., aged twenty. Badly broken down superior right lateral incisor, but pulp vital. Excised the remaining portion of the crown with the aid of fissure bur and forceps preparatory to crowning, leaving a very small exposure. Applied cocaine and alcohol, and extracted pulp with no pain at all.

*November 16.*—Master B., aged twelve. Badly aching inferior left first molar, occlusal cavity, small exposure. Cocaine and alcohol were applied, which permitted of exposing the pulp more freely, when with a second application it was extracted with very slight pain.

*November 18.*—Mrs. L., aged about forty-five. Inferior left first bicuspid, distal cavity. Arsenic had been applied the day preceding, and now the extraction of the pulp was attempted, but it was found just as sensitive as twenty-four hours previously. Cocaine and alcohol were applied, and the pulp was extracted with comparatively no pain.

*November 18.*—Mrs. H., aged twenty-four. Inferior left first bicuspid, mesial cavity, pulp exposed and aching. Arsenic had been applied six months before and again removed, leaving the pulp still vital and very sensitive. Cocaine and alcohol were used, the pulp was extracted without pain, and the canal dressed; it was filled at a subsequent sitting with oxychloride of zinc, and the crown cavity with gold.

*November 20.*—Mr. E., aged seventeen, very nervous. Superior right first bicuspid, cavity involving mesial and distal surfaces, pulp exposed. Cocaine and alcohol were applied, and the pulp was removed painlessly.

*November 22.*—Mr. P., aged twenty-three. Superior left second bicuspid, distal cavity; tooth treated about three weeks previously, patient thought for devitalization; pulp quite sensitive. Applied cocaine and alcohol, and extracted with very slight pain.

*November 25.*—Miss M., aged eighteen. Superior right second bicuspid, broken down almost to the gum, pulp very sensitive. Applied cocaine and alcohol, and extracted painlessly. Tooth crowned.

*November 25.*—Miss F., aged twenty. Superior left first molar, occlusal cavity. Applied cocaine and alcohol, and opened pulp-chamber with a bur, removing the bulbous portion, but could not enter canals; after second application the pulp was removed from the palatine canal without pain, but before the buccal canals could be entered a third treatment was necessary, and even then there was slight pain, but not sufficient to prevent their being entered and cleansed.

*November 27.*—Mrs. L., aged twenty-seven. Superior right lateral incisor, mesial surface. Pulp exposed in excavating, but too shallow to permit of proper capping and filling with gold, which latter had been determined on. Applied cocaine and alcohol, and extracted pulp with very slight pain. After treating properly the canal was filled at the same sitting with oxychloride of zinc, and the crown cavity at a subsequent sitting with gold.

*December 5.*—Mr. S., aged twenty-seven. Inferior left second molar, occlusal cavity, and extending down over the buccal surface to the gum margin. Applied cocaine and alcohol, and extracted pulp; no pain whatever except during pressure on the rubber, which was very slight.

*December 5.*—Miss B., aged eighteen. Superior right second bicuspid, cavity on mesial surface; very slight exposure of the buccal horn. Applied cocaine and alcohol, and with a bur exposed pulp more freely and made a second application, when the pulp was extracted in its entirety with very slight pain. Canal filled later with oxychloride of zinc, and crown cavity with gold.

*December 7.*—Mrs. L., aged thirty-eight. Superior right cuspid, cavity in distal surface. Applied cocaine and alcohol, and extracted pulp with no pain. Time required to anæsthetize and extirpate pulp, just *two minutes.*

*December 8.*—Mr. A., aged seventeen. Superior right first bicuspid, cavity on distal surface, extending well to the cervix. Two days before, December 6, the patient had applied for treatment; the tooth was very sensitive on excavating, so a thin layer of decayed tissue was left over the pulp and oil of cinnamon sealed in the cavity. Patient returned next day, having suffered the greater part of the night, during which time oil of cloves had brought temporary relief. On opening the cavity it was found extremely sensitive, so carbolic acid was sealed in and the patient asked to call next day, the intention being to apply arsenic after relieving the pulp of its inflamed condition. On presenting, the conditions were about the same. What tissue remained over the pulp was removed, exposing it; an application of cocaine and alcohol was made and pulp exposed more freely with a bur, and after a second application extracted, *with absolutely no pain.* This case took about eight or ten minutes.

*December 10.*—Mr. E., aged thirty-two, had applied one week previously; the superior right first bicuspid, distal surface, contained a badly leaking amalgam filling, and the tooth was aching. The filling was removed and an exposure found, which was treated with carbolic acid and iodoform sealed in the cavity, thinking the pulp partly dead, and that after a week its removal would be quite easy. On returning December 10, however, it was quite sensitive, so much so that excavation of cavity was impossible. Cocaine and alcohol were applied, and pulp was extracted from both canals with very slight pain. The canals were subsequently filled with oxychloride of zinc and the tooth refilled with amalgam.

*December 13.*—Mr. M., aged thirty-two. Inferior right first molar, mesial cavity, extending very far under the gum; small exposure. Made a number of attempts with cocaine and alcohol, cocaine, alcohol, and formalin, and cocaine and vapocaine, but each on pressure produced extreme pain, which continued. After persevering for about a half-hour or more, the pulp finally submitted and the canals could be entered painlessly, but in this case the time required and the agony produced would not seem to render the treatment justifiable.

*January 6.*—Mr. C., aged thirty. Inferior right second molar. On October 31 a very shallow cavity was filled on the occlusal surface with amalgam. About November 14 the tooth began to give trouble, which continued until December 5, when the filling was removed and treatment commenced to relieve the pulpitis, which was continued till December 12 without effect. It was then decided to open into the pulp-chamber and devitalize. After exposing the pulp an attempt was made to relieve the congestion preparatory to devitalization with arsenic. The pain did not seem to be relieved, however, and the pulp began to puff up through the exposed area, and had all the characteristics of a fungoid pulp, when, on January 6, it was decided to extract it with the cocaine method. After a single application the chamber was opened with a bur and the pulp removed from both canals without any pain. The canals were subsequently filled with oxychloride of zinc, and the cavity with gold.

*January 11.*—Miss R., aged seventeen. Inferior left first molar, cavity on mesial and occlusal surfaces. Three days previously, while excavating, there appeared a very slight exposure, which was treated with acetate of morphia and carbolic acid preparatory to capping, with instructions to patient to return in three days. On her return there had been a great deal of aching, so pulp extirpation with cocaine and alcohol was determined upon. After first application the pulp-chamber could be opened and the bulbous portion removed with a bur, but the canals could not be entered. Two other applications were made, one to the entrance of each canal, and all tissue was removed with practically no pain.

*January 12.*—Miss P., aged twenty-three, very nervous. Inferior left second bicuspid, distal cavity. Cocaine and alcohol were applied as usual; on first pressure considerable pain was produced, which left very suddenly, and after about one minute the chamber was freely opened with a bur and pulp extracted with no further pain.

Same patient four days later. Exposure in superior left lateral incisor, mesial surface. Same treatment was attempted, but on slightest pressure intense pain was produced, which would not subside; the operation was tried repeatedly, with the same result, till at last the patient became too nervous and refused further attempts; finally after some persuasion she submitted to one more trial, with the assurance that it should be the last; the pain seemed as intense as before when pressure was applied, but suddenly it began to grow less and soon ceased entirely, when the pulp was extracted without further trouble.

*January 15.*—Miss B. M., aged twenty. Superior left first bicuspid, distal surface. After one application the pulp was extracted, although with some little pain.

*January 16.*—Miss S., aged twenty-two. Inferior right second molar, cavity occlusal, exposure small. After one application the pulp was removed without any pain. Canals were filled one week later with oxychloride of zinc, and the cavity with amalgam.

*January 17.*—Miss L., aged twenty-four. Superior left second bicuspid, mesial cavity. Made five or six trials, but each proved so painful that further attempt was abandoned and arsenic resorted to.

*January 18.*—Miss H., aged twenty. Superior left second bicuspid, distal cavity. Made one application and removed pulp without pain.

*January 19.*—Miss D., aged sixteen. Inferior right first molar, occlusal surface; had been treated three days previously with oil of cloves to relieve toothache. After one application of cocaine and alcohol the pulp was removed with very slight pain.

*January 19.*—Miss C., aged twenty-eight. Inferior left third molar, occlusal surface, large exposure, aching badly. After one application the pulp was removed without any pain.

*January 19.*—Miss E. M., aged thirty-eight. Inferior right first bicuspid, distal surface; exposure at the cervical portion of a leaking cement filling. Three days before an application of arsenic, carbolic acid, and iodoform was made, but on attempt at removal of pulp it was as much alive, so far as sensation was concerned, as three days before, so cocaine treatment was used and pulp removed with no pain.

*January 25.*—Miss K., aged seventeen. Superior right first bicuspid, distal cavity. Oil of cloves had been sealed in two days before, giving entire relief, although previously it had ached badly. After one application of cocaine and alcohol the pulp was removed from both canals absolutely painlessly, although coming out in shreds.

*February 6.*—Miss C., aged eighteen, very nervous. Inferior left second molar, occlusal exposure. Made a number of attempts with cocaine and alcohol, but the pain on pressure was so great and continued that the treatment had to be abandoned as a complete failure in this case.

*February 13.*—Miss I., aged seventeen. Superior left first bicuspid, distal surface, pulp congested. One application. No pain at all upon extraction.

*February 13.*—Miss O., aged eighteen. Inferior left second molar, mesial cavity. Required three applications, but was extracted almost painlessly.

*March 1.*—Mrs. L., aged forty. Inferior right first bicuspid, distal cavity, pulp exposed and bleeding during excavating. Applied cocaine and alcohol, and extracted pulp with very slight pain.

*March 21.*—Miss M., aged twenty. Superior left second bicuspid, cavity including mesial, distal, and occlusal surfaces; slight mesial exposure, pulp congested. After first application the exposure was enlarged and a second treatment applied, after which the pulp was extracted with no pain at all.

This makes a collection of thirty-eight cases given in the order in which they presented, accepting the conditions as they appeared, without any attempt to shirk apparently unfavorable cases.

I shall not pretend to say, but leave it to your judgment, whether or not from the list of conditions given, together with the results obtained, the treatment is worthy of being advocated. To myself it has proved eminently satisfactory.

I should like to append to the above a few words on a method we have more recently been using in an experimental way to anæsthetize pulps, which is the injection of chloroform with the hypodermic syringe, using a very fine needle. By bringing the needle point just to the exposed tissue and immediately forcing a drop of chloroform on to the tissue, at the same instant also forcing the needle point into it, together with additional chloroform. The needle may in this way be forced with little or no pain well into it, when, after a few drops of additional chloroform have been injected, the pulp can instantly be extracted without the slightest sensation. It requires no waiting after injecting, and all appliances should previously be prepared, such as the engine with bur to open the pulp-chamber freely if necessary, and whatever extractors or broaches are to be used.

I have recently very successfully taken pulps from incisors, cuspids, bicuspids, and molars without regard to the condition of pulps; also where any sensitive tissue has been left in root-canals with the rest of the pulp dead and perhaps removed, if the needle point can be inserted and a few drops of chloroform forced in, the previously sensitive tissue can be extracted without any pain, while previously perhaps the sensitiveness was so great as to make entrance with a broach impossible. I am using this method quite extensively at present, although time has not permitted the gathering of data to be presented herewith.

Mention of it is made at this time with the hope that some of those present may try it and find it in very many cases even preferable to the cocaine and pressure method. Where a patient presents with an exposed pulp and a violent toothache, the pain can be almost instantly relieved by this treatment, and after the injection the pulp can be extracted without even the slightest sensation.

